# Progress Toward Measles Elimination — Western Pacific Region, 2009–2012

**Published:** 2013-06-07

**Authors:** W. William Schluter, Wang Xiaojun, Jorge Mendoza-Aldana, Youngmee Jee, Sergey Diorditsa, Alya Dabbagh, Mick Mulders, Christopher Gregory, James L. Goodson

**Affiliations:** Expanded Programme on Immunization, World Health Organization Western Pacific Regional Office, Manila, Philippines; Dept of Immunization, Vaccines, and Biologicals, World Health Organization, Geneva, Switzerland. Div of Viral Diseases, National Center for Immunization and Respiratory Diseases; Global Immunization Div, Center for Global Health, CDC

In 2005, the World Health Organization (WHO) Regional Committee for the Western Pacific Region (WPR) resolved that WPR should aim to eliminate measles* by 2012 (1). The recommended measles elimination strategies (2) in WPR include 1) achieving and maintaining high (≥95%) coverage with 2 doses of measles-containing vaccine (MCV) through routine immunization services and by implementing supplementary immunization activities (SIAs), when required; 2) conducting high-quality, case-based measles surveillance; 3) ensuring high-quality laboratory surveillance, with timely and accurate testing of specimens to confirm or discard suspected cases and detect measles virus for genotyping and molecular analysis; and 4) establishing and maintaining measles outbreak preparedness for rapid response and ensuring appropriate case management. This report updates the previous report (3) and describes progress toward eliminating measles in WPR during 2009–2012. During this period, measles incidence reached a historic low, decreasing by 83%, from 34.0 to 5.9 cases per million population. However, to achieve measles elimination in WPR, additional efforts are needed to strengthen routine immunization services in countries and areas with <95% coverage with the routine first (MCV1) or second dose of MCV (MCV2), to introduce a MCV2 dose in the four remaining countries and areas that do not yet have a routine 2-dose MCV schedule, and to use SIAs to close immunity gaps among measles-susceptible populations in countries and areas that have ongoing measles virus transmission.

## Immunization Activities

Annual data on MCV coverage are reported from 36 of the 37 WPR countries and areas to WHO and the United Nations Children’s Fund (UNICEF).† MCV1 coverage in WPR increased from 96% in 2009 to 98% in 2012. The number of countries with ≥95% MCV1 coverage increased from 12 (33%) in 2009 to 15 (42%) in 2012. MCV1 was administered at 8 months in one (3%), at age 9 months in six (17%),§ at age 10 months in one (3%), at age 12 months in 24 (67%), and at age >12 months in four (11%) (Table 1).

The number of countries and areas that provide routine MCV2 increased from 32 (89%) in 2009 to 33 (92%) in 2012, and the number reporting ≥95% MCV2 coverage increased from 10 (28%) in 2009 to 11 (31%) in 2012. Among the 33 countries and areas reporting MCV2 coverage in 2012, the scheduled age of MCV2 administration ranged from 12 months to 7 years. During 2009–2012, approximately 226 million children were vaccinated during 16 measles SIAs (Table 2); of these, seven (44%) SIAs included rubella vaccine, and 10 (63%) added at least one other child health intervention.

## Surveillance Activities

During 2009–2012, measles case-based surveillance was conducted in all 37 WPR countries and areas, including 14 countries and two areas that report data individually, and 21 countries and areas of the Pacific Islands that report data as one epidemiologic block.¶ Measles surveillance data are reported monthly to WHO and supported by 385 laboratories participating in the WHO Global Measles and Rubella Laboratory Network** (4). Suspected measles cases were confirmed based on laboratory findings, an epidemiologic link, or clinical criteria.†† Key indicators of surveillance performance include 1) the number of suspected measles cases discarded as nonmeasles (target: ≥2 per 100,000 population); 2) the proportion of second-level administrative units with ≥1 nonmeasles discarded case per 100,000 population (target: ≥80%); 3) the percentage of suspected measles cases with adequate investigation that includes all essential data elements§§ (target: ≥80%); 4) the percentage of suspected measles cases with adequate specimens collected within 28 days of rash onset (target: ≥80%, excludes epidemiologically linked cases) (5); and 5) the percentage of specimens with laboratory results available within 7 days after receipt in the laboratory (target: ≥80%). The number of countries and areas with adequate data that met the target for suspected cases discarded as nonmeasles per 100,000 population increased from seven (50%) of 14 in 2009 to nine (64%) of 14 in 2012 (Table 3). From 2009 to 2012, suspected cases with adequate investigations increased from 38% to 89%, suspected cases with adequate specimens collected for laboratory testing increased from 79% to 93%, and the proportion of blood specimens received by the laboratory with results available within 7 days increased from 55% to 96% (Table 3).

## Measles Disease Incidence and Measles Virus Genotypes

From 2009 to 2012, confirmed measles cases decreased 84%, from 54,291 to 8,524, and confirmed measles incidence per million population decreased 83%, from 34.0 to 5.9 (Table 1). In 2012, the highest confirmed measles incidence was reported from Malaysia (63.7 per million), the Philippines (15.9 per million), and New Zealand (12.3 per million) (Table 1). The highest number of confirmed cases was reported from China and decreased 88%, from 52,461 in 2009 to 6,183 in 2012 (Figure). During 2009–2012, the predominant measles virus genotypes detected in WPR were H1 in China, D9 in the Philippines, Malaysia, and Singapore; and D8 in Malaysia. Other measles virus genotypes that were identified and determined to have been related to measles virus importations from outside WPR included B3, D4, and G3.

What is already known on this topic?

The World Health Organization (WHO) Regional Committee for the Western Pacific Region (WPR) has resolved to eliminate measles by 2012. Substantial progress had been made in reducing the burden from measles by most countries in the region by 2008. The number of reported measles cases in WPR (excluding China) decreased 86%, from 106,172 (255.6 per million population) in 2000 to 14,724 (32.6 per million population) in 2008.

What is added by this report?

This report updates the previous report that summarized progress during 1990–2008 and describes progress toward measles elimination in WPR during 2009–2012. During this period, measles incidence in the region reached a historic low, decreasing by 83%, from 34.0 to 5.9 cases per million population. In China, a nationwide measles vaccination campaign was implemented in 2010 and reported confirmed measles cases decreased 88%, from 52,461 in 2009 to 6,183 in 2012.

What are the implications for public health practice?

Despite the progress to date, achieving measles elimination in WPR will require additional efforts. These include 1) introducing a routine second dose of measles-containing vaccine (MCV) in the four remaining countries and areas that do not yet have a routine 2-dose MCV schedule; 2) strengthening routine immunization services in countries and areas with <95% coverage with the routine first or second dose of MCV; and 3) closing immunity gaps through supplementary immunization activities in measles-susceptible populations in countries and areas that have ongoing measles virus transmission.

### Editorial Note

In 2012, the WPR Regional Committee reaffirmed its commitment to eliminate measles and urged member states to interrupt all residual endemic measles virus transmission as rapidly as possible (6). To achieve elimination, intensified efforts are needed to identify and close gaps in population immunity, by increasing coverage with MCV2 to ≥95% in all countries and areas and by conducting high-quality SIAs in countries with sustained measles virus transmission (e.g., China, Malaysia, and the Philippines). In countries and areas with <95% MCV1 or MCV2 coverage, urgent action is needed to strengthen routine immunization services and to identify and implement targeted SIAs for measles-susceptible populations. In the four remaining countries and areas (Lao People’s Democratic Republic, Papua New Guinea, Solomon Islands, and Vanuatu) that do not provide MCV2 in the routine childhood vaccination schedule, strategies are needed to increase MCV1 coverage, conduct periodic SIAs to provide a second opportunity for all birth cohorts to receive MCV, and prepare for introduction of routine MCV2.

The WPR *Guidelines on Verification of Measles Elimination* (7) were finalized in March 2013; progress toward measles elimination in WPR will be monitored by the Regional Verification Commission through annual progress reports from each country or area and from the Pacific Islands countries and areas reporting as one epidemiologic block. High-quality case-based measles surveillance is critical to the verification process. Despite overall improvement in measles surveillance performance, gaps persist, as reflected by the low proportion of second-level administrative units with one or more nonmeasles discarded case per 100,000 population. Additionally, incomplete investigations of suspected measles cases in some countries challenge efforts to rapidly identify and respond to outbreaks and to measure and document progress toward elimination. For example, in Vietnam, only six (0.8%) of the 771 suspected measles cases with specimens available for testing reported in 2012 were laboratory confirmed. However, 631 additional cases did not have specimens collected but were reported as clinically confirmed measles. The sensitivity of the measles surveillance system in other countries with discarded nonmeasles reporting rates of <2 per 100,000 population might be insufficient to rapidly detect and respond to outbreaks or to meet verification criteria.

The WHO *Global Vaccine Action Plan* calls for the elimination of rubella and congenital rubella syndrome in five of the six WHO regions by 2020 (8). In April 2012, the Measles and Rubella Initiative launched the 2012–2020 Global Measles and Rubella Strategic Plan to integrate rubella with measles elimination efforts (9). Rubella-containing vaccine is not provided in six WPR countries and areas; five of these countries (Cambodia, Lao People’s Democratic Republic, Papua New Guinea, Solomon Islands, and Vietnam) are eligible for financial support offered by the GAVI Alliance to conduct a wide-age-range SIA using combined measles-rubella vaccine followed by the introduction of rubella vaccine in their national routine immunization programs. In addition to contributing to rubella elimination, these SIAs would provide a unique opportunity to boost population immunity to measles and contribute momentum to achieve and sustain measles elimination in WPR.

## Figures and Tables

**FIGURE:**
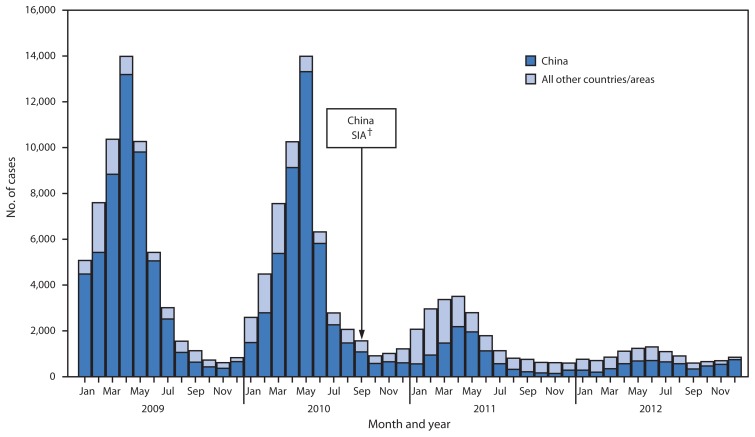
Confirmed measles cases,* by month of rash onset — World Health Organization Western Pacific Region (WPR), 2009–2012 **Abbreviation:** SIA = supplementary immunization activity. * Confirmed measles cases reported by countries and areas to World Health Organization. A case of measles is confirmed by serology when measles-specific immunoglobulin M antibody is detected in a person who was not vaccinated in the previous 30 days. A case of measles is confirmed by epidemiologic linkage when linked in time and place to a laboratory-confirmed measles case but lacks serologic confirmation. During 2009–2012, a case of measles meeting the case definition but without a specimen collected could be reported as clinically confirmed. ^†^ SIA conducted in China in which approximately 100 million children aged 8–179 months were vaccinated against measles, with targeted age group varying by province.

**TABLE 1:** Reported coverage with the first and second dose of measles-containing vaccine (MCV),* age of vaccination, number of confirmed measles cases, and confirmed measles incidence, by country/area — World Health Organization Western Pacific Region, 2009 and 2012

Country/Area	2009						2012					
	
	% coverage with the first MCV dose	% coverage with the second MCV dose	Country or area MCV schedule†		No. of confirmed measles cases	Measles incidence per million population	% coverage with the first MCV dose	% coverage with the second MCV dose	Country or area MCV schedule		No. of confirmed measles cases	Measles incidence per million population
	
			1st dose	2nd dose					1st dose	2nd dose		
American Samoa	NR§	NR	M12	Y4	0	0.0	NR	NR	M12	Y4	0	0.0
Australia	94	83	Y1	Y4	104	5.0	94	91	M12	Y4	199	8.7
Brunei Darussalam	100	99	Y1	Y3	2	5.0	99	96	Y1	Y3	1	2.4
CNMI	87	84	M12	Y4	0	0.0	68	65	M12	Y4–6	0	0.0
Cambodia	92	NA¶	M9–11	NA	865	58.9	93	82	M9	M18	0	0.0
China	99	98	M8	M18–24	52,461	39.5	100	100	M8	M18	6,183	4.6
Cook Islands	78	61	M15	Y4	0	0.0	97	98	M15	Y4	0	0.0
Fiji	72	57	M12	Y6	4	1.3	90	NR	M12	Y6	0	0.0
French Polynesia	99	84	M12	M24	0	0.0	NR	99	M10	M15	0	0.0
Guam	NR	NR	M12	Y4–6	0	0.0	51	44	Y1	Y4–6	0	0.0
Hong Kong (China)	98	99	M12	P1	22	3.1	96**	98	M12	P1	8	1.1
Japan	94	92	Y1	Y5	705	5.5	95	93	Y1	Y5	228	1.8
Kiribati	82	35	Y1	Y6	0	0.0	91	61	M12	P1	0	0.0
Lao People’s Democratic Republic	59	NA	M9	NA	72	12.1	72	NA	M9	NA	36	5.6
Macao (China)	91	88	M12	M18	0	0.0	93	89	M12	M18	1	1.8
Malaysia	95	95	Y1††	Y7	56	2.1	86	99	Y1††	Y7	1,868	63.7
Marshall Islands	78	66	M12	M13	0	0.0	78	58	M12	M15	0	0.0
Micronesia	86	82	M12	M13	0	0.0	91	70	M12	M13	0	0.0
Mongolia	94	97	M9	Y2	8	3.0	99	98	M9	Y2	0	0.0
Nauru	100	92	M12	M15	0	0.0	96	81	M12	M15	0	0.0
New Caledonia	99	78	M12	Y2	0	0.0	96	86	M12	Y2	0	0.0
New Zealand	89	NR	M15	Y4	253	60.0	92	85	M15	Y4	55	12.3
Niue	100	100	M15	Y4	0	0.0	100	98	M15	Y4	0	0.0
Palau	75	NR	M12	M15	0	0.0	91	86	M12	M15	0	0.0
Papua New Guinea	58	NA	M9§§	NA	0	0.0	67	NA	M9§§	NA	0	0.0
Philippines	88	58¶¶	M9	M12–15	1,490	16.6	85**	38**	M9	M12–15	1,536	15.9
Republic of Korea	93	100	M12–15	Y4–6	17	0.4	99	97	M12–15	Y4–6	2	0.0
Samoa	49	29	M12	M15	0	0.0	85	67	M12	M15	0	0.0
Singapore	95	93	Y1–2	Y6–7	16	3.6	NR	NR	M12	M15–18	40	7.6
Solomon Islands	60	NA	M12	NA	0	0.0	85	NA	M12	NA	0	0.0
Tokelau	100	100	M12	M15	0	0.0	100	85	M12	M15	0	0.0
Tonga	99	98	M12	M18	0	0.0	95	95	M12	M18	0	0.0
Tuvalu	90	84	M12	M18	0	0.0	98	93	M12	M18	0	0.0
Vanuatu	80	NA	Y1	NA	0	0.0	94	NA	Y1	NA	0	0.0
Vietnam	97	96	M9	Y6	5,222	59.0	96	83	M9	M18	637	7.1
Wallis and Futuna Islands	NR	NR	M9	M18	0	0.0	120	107	M12	M18	0	0.0
**Western Pacific Region**	**96**	**94**			**54,291**	**34**	**98**	**97**			**8,524**	**5.9**

**Abbreviation:** CNMI = Commonwealth of the Northern Mariana Islands.

* Country or area reported coverage for first or second dose of MCV based on administrative data or coverage survey data, if available.

† Country MCV schedule abbreviations: M = month of age when dose is given; Y = years of age when dose is given; and P = primary grade of school when dose is given.

§ NR = not reported (country did not report coverage in the year specified).

¶ NA = not applicable (dose was not included in the vaccination schedule for that year).

** Data are preliminary.

†† Additional 6-month dose provided subnationally.

§§ Additional 6-month dose provided nationally.

¶¶ Second dose administered at subnational level; therefore, the denominator is from the population served only.

**TABLE 2:** Characteristics of measles supplementary immunization activities (SIAs),* by year and country/area — World Health Organization Western Pacific Region, 2009–2012

Year	Country/Area	Age group targeted (mos)	Measles- containing vaccine used	Children reached in targeted age group		Other interventions delivered			
	
				No.	(%)	Oral polio vaccine	Vitamin A	Deworming medication	Tetanus toxoid vaccination
2009	China	8–179†	M	94,167,415	(98)				
	Kiribati	12–59	MR	9,865	(106)		Yes	Yes	
	Papua New Guinea	6–83	M	945,582	(86)				
	Solomon Islands	12–59	M	60,025	(90)				
	Vanuatu	12–59	M	29,919	(97)				
2010	China	8–179†	M	102,300,000	(97)				
	Federated States of Micronesia	12–83	MMR	11,485	(90)		Yes	Yes	
	Papua New Guinea	6–35	M	464,973	(83)	Yes	Yes	Yes	
	Tuvalu	12–71	MR	1,095	(79)		Yes	Yes	
	Vietnam	9–71	M	7,034,895	(96)				
2011	Cambodia	9–119	M	1,819,360	(100)	Yes	Yes	Yes	Yes
	Lao People’s Democratic Republic	9–228	MR	2,614,002	(97)	Yes	Yes	Yes	
	Philippines	9–95	MR	15,649,907	(84)	Yes			Yes
2012	Mongolia	36–179	MR	522,414	(91)	Yes			
	Papua New Guinea	6–35	M	552,872	(88)	Yes	Yes	Yes	Yes
	Solomon Islands	12–59	MR	68,261	(102)		Yes	Yes	
**2009–2012**	**Western Pacific Region**			**226,252,070**	**(96)**				

**Abbreviations:** M = measles vaccine; MR = measles and rubella vaccine; MMR = measles, mumps, and rubella vaccine.

* SIAs generally are carried out using two approaches. An initial, nationwide catch-up SIA targets all children aged 9 months–14 years; it has the goal of eliminating susceptibility to measles in the general population. Periodic follow-up SIAs then target all children born since the last SIA. Follow-up SIAs generally are conducted nationwide every 2–4 years and generally target children aged 9–59 months; their goal is to eliminate any measles susceptibility that has developed in recent birth cohorts and to protect children who did not respond to the first measles vaccination. The exact age range for follow-up SIAs depends on the age-specific incidence of measles, coverage with measles-containing vaccine through routine services, and the time since the last SIA.

† Targeted age groups varied by province.

**TABLE 3:** Measles surveillance indicators and targets, by country, area, or epidemiologic block* — World Health Organization, Western Pacific Region, 2009 and 2012

Country, area, or epidemiologic block	2009					2012				
	
	Discarded nonmeasles rate per 100,000	Second-level units with ≥1 discarded cases per 100,000	Suspected cases with adequate investigation	Suspected cases with adequate blood specimens†	Laboratory results in ≤7 days of specimen reception	Discarded nonmeasles rate per 100,000	Second-level units with ≥1 discarded cases per 100,000	Suspected cases with adequate investigation	Suspected cases with adequate blood specimens†	Laboratory results in ≤7 days of specimen reception
*Target*	*≥2*	*≥80%*	*≥80%*	*≥80%*	*≥80%*	*≥2*	*≥80%*	*≥80%*	*≥80%*	*≥80%*
Australia§	ID ¶	ID	ID	ID	100.0	ID	ID	ID	ID	100.0
Brunei Darussalam	1.5	100.0	75.0	75.0	NA**	1.5	100.0	71.4	85.7	NA
Cambodia	26.4	58.3	62.0	98.4	38.7	6.8	58.3	56.1	99.2	98.3
China	1.3	54.8	86.9	70.1	76.2	2.3	71.0	99.0	97.9	97.1
Hong Kong (China)	0.1	100.0	46.9	71.9	96.2	2.5	100.0	92.0	97.3	98.7
Macao (China)	3.7	100.0	100.0	100.0	98.2	3.9	100.0	95.7	100.0	96.6
Japan	0.0	0.0	ID	ID	ID	0.1	0.0	ID	ID	ID
Lao People’s Democratic Republic	2.5	35.3	57.8	60.0	94.0	7.6	64.7	49.3	76.6	93.7
Malaysia	7.9	86.7	34.1	72.4	100.0	22.7	93.8	74.4	83.4	97.7
Mongolia	6.4	47.6	34.5	98.9	100.0	22.0	40.9	64.2	100.0	100.0
New Zealand	ID	ID	ID	ID	99.5	ID	ID	ID	ID	99.3
Papua New Guinea	1.2	15.0	26.8	2.4	NA	0.6	10.0	61.9	81.0	57.6
Philippines	1.6	82.4	29.4	73.8	73.5	2.1	64.7	56.5	79.4	95.3
Republic of Korea	0.1	0.0	40.3	62.7	96.1	0.3	6.3	84.0	90.4	100.0
Singapore	ID	ID	ID	ID	96.4	ID	ID	ID	ID	96.9
Vietnam	4.5	78.1	27.5	72.4	42.5	0.9	25.0	44.3	55.0	96.6
Pacific Islands countries and areas	2.6	13.0	9.9	14.3	100.0	5.7	ID	0.0	ID	93.4
**Western Pacific Region**	**2.8**	**43.1**	**38.0**	**78.8**	**54.9**	**2.4**	**35.1**	**88.8**	**93.1**	**96.0**

* The 21 Pacific Islands countries and areas are considered as one epidemiologic block for purposes of measles surveillance.

† Excludes epidemiologically linked cases.

§ Reports only confirmed cases.

¶ ID = Insufficient data reported by the country to calculate the indicator.

** NA = not available; no World Health Organization–accredited laboratory in the country.

## Footnotes

* Measles elimination is defined as the absence of endemic measles virus transmission in a defined geographic area (e.g., region or country) for ≥12 months in the presence of a well-performing surveillance system.

† The Pitcairn Islands, with a population of approximately 50 persons, does not report immunization coverage data to WHO/UNICEF.

§ Papua New Guinea also provides a supplementary dose of MCV at age 6 months.

¶ The epidemiologic block of the Pacific Islands countries and areas includes American Samoa, Cook Islands, Fiji, French Polynesia, Guam, Kiribati, the Marshall Islands, the Federated States of Micronesia, Nauru, New Caledonia, Niue, the Commonwealth of the Northern Mariana Islands, Palau, the Pitcairn Islands, Samoa, Solomon Islands, Tokelau, Tonga, Tuvalu, Vanuatu, and Wallis and Futuna.

** This network includes one WHO global specialized laboratory in Japan, three regional reference laboratories (in Melbourne, Australia; Beijing, China; and Hong Kong, China), 19 national or subnational laboratories, and 31 provincial and 331 prefecture-level laboratories in China.

†† Cases that meet the WHO clinical case definition of measles for which no adequate specimen was collected and cannot be epidemiologically linked to a laboratory-confirmed case of measles.

§§ Essential data elements include name or identifier, date of birth or age, sex, place of residence, vaccination status or date of last vaccination, date of rash onset, date of notification, date of investigation, date of specimen collection, and place of infection or travel history.
